# Comparative Proteomic Analysis of Adhesion/Invasion Related Proteins in *Cronobacter sakazakii* Based on Data-Independent Acquisition Coupled With LC-MS/MS

**DOI:** 10.3389/fmicb.2020.01239

**Published:** 2020-06-09

**Authors:** Ping Li, Xuan Dong, Xiao-yi Wang, Ting Du, Xin-jun Du, Shuo Wang

**Affiliations:** ^1^State Key Laboratory of Food Nutrition and Safety, Tianjin University of Science and Technology, Tianjin, China; ^2^Tianjin Key Laboratory of Food Science and Health, School of Medicine, Nankai University, Tianjin, China

**Keywords:** *C. sakazakii*, adhesion/invasion capability, sequence type, DIA proteomic, real-time qPCR

## Abstract

*Cronobacter sakazakii* is foodborne pathogen that causes serious illnesses such as necrotizing enterocolitis, meningitis and septicemia in infants. However, the virulence determinants and mechanisms of pathogenicity of these species remain unclear. In this study, multilocus sequence typing (MLST) was performed on 34 *C. sakazakii* strains and two strains with the same sequence type (ST) but distinct adhesion/invasion capabilities were selected for identification of differentially expressed proteins using data-independent acquisition (DIA) proteomic analysis. A total of 2,203 proteins were identified and quantified. Among these proteins, 210 exhibited differential expression patterns with abundance ratios ≥3 or ≤0.33 and *P* values ≤0.05. Among these 210 proteins, 67 were expressed higher, and 143 were expressed lower in *C. sakazakii* SAKA80220 (strongly adhesive/invasive strain) compared with *C. sakazakii* SAKA80221 (weakly adhesive/invasive strain). Based on a detailed analysis of the differentially expressed proteins, the highly expressed genes involved in flagellar assembly, lipopolysaccharide synthesis, LuxS/AI-2, energy metabolic pathways and iron-sulfur cluster may be associated with the adhesion/invasion capability of *C. sakazakii*. To verify the accuracy of the proteomic results, real-time qPCR was used to analyze the expression patterns of some genes at the transcriptional level, and consistent results were observed. This study, for the first time, used DIA proteomic to investigate potential adhesion/invasion related factors as a useful reference for further studies on the pathogenic mechanism of *C. sakazakii*.

## Introduction

*Cronobacter* species are rod-shaped, peritrichous, motile, Gram-negative, non-spore-forming facultative anaerobes that are widely present in nature (Iversen and Forsythe, 2003). The genus *Cronobacter* has been classified into seven species, namely, *Cronobacter sakazakii*, *Cronobacter malonaticus*, *Cronobacter turicensis*, *Cronobacter condimenti*, *Cronobacter muytjensii*, *Cronobacter dublinensis*, and *Cronobacter universalis* (Iversen et al., 2007, 2008; Joseph et al., 2012; Stephan et al., 2014). Genome analysis has shown that only *C. sakazakii* and *C. malonaticus* are associated with severe infections (Forsythe, 2018). *Cronobacter* spp. can cause necrotizing enterocolitis (NEC), sepsis and meningitis in newborns, low-birth-weight infants (<2.5 kg), immunocompromised newborns and infants under 4 weeks of age with a mortality rate of approximately 27% (Lai, 2001; Friedemann, 2009; Holy and Forsythe, 2014). Furthermore, *Cronobacter* species can also infect elderly and immunocompromised adults, leading to bacteremia, osteomyelitis, splenic abscess, pneumonia, conjunctivitis, wound infections, and urinary tract infections (Patrick et al., 2014; Blackwood and Hunter, 2016).

Given the adverse conditions associated with *Cronobacter*, *in vitro* studies utilizing human-derived cell lines as well as *in vivo* studies utilizing suckling mice or rats have been employed to explore the mechanism of adhesion/infection of *Cronobacter* spp. (Townsend et al., 2007; Giri et al., 2012; Quintero-Villegas et al., 2014). Several studies using gene knockouts have demonstrated that outer membrane protein A (OmpA), outer membrane protein X (OmpX) and outer membrane protease Cpa as well as the gene *bcsR* are crucial virulence factors of *Cronobacter* (Nair et al., 2009; Kim et al., 2010; Franco et al., 2011; Singh et al., 2015; Gao et al., 2017). Additionally, it has been demonstrated that enterotoxins, lipopolysaccharides (LPSs) and flagella are closely associated with the virulence of *Cronobacter* (Hotta et al., 1986; Pagotto et al., 2003; Jing et al., 2016). Although the above mentioned genes and factors are relevant for the virulence of *Cronobacter*, little is known regarding the functions and fundamental mechanisms of other virulence determinants in comparison with those of other major foodborne pathogens. Therefore, further studies on the pathogenesis of *Cronobacter* are necessary to understand these bacteria and to establish effective strategies for prevention and therapy.

Proteomic techniques are useful tools for investing the mechanisms underlying certain phenotypes or characteristics, such as virulence, desiccation tolerance, and osmotic tolerance (Jiang et al., 2007). Two-dimensional electrophoresis (2-DE) protein analysis couple with MALDI-TOF MS was used to investigate changes in the protein patterns of *Enterobacter sakazakii* cells in response to osmotic stress, and the result indicated that the genes *rpoS* and *ompC* played vital roles in osmotic shock (Riedel and Lehner, 2007). In our previous study, 2-DE was employed to identify virulence-related proteins. A total of 89 proteins were successfully identified and further analysis suggested that 11 of these proteins may be involved in the virulence of this pathogen (Du et al., 2015). Although 2-DE is a powerful proteomics approach for screening and identification of diagnostic markers and therapeutic targets in pathogenic microbes, there are also limitations associated with this technique, such as instability, complexity, and low sensitivity (Orsatti et al., 2009). Iso-baric tags for relative and absolute quantitation (iTRAQ) was used to study the proteome of *C. sakazakii* cells under dessication. The results indicated 233 differentially expressed proteins involved in trehalose and betaine uptake, which were associated with the ability of *C. sakazakii* ATCC 29544 to withstand osmotic stress (Hu et al., 2017). Nevertheless, LC-MS/MS coupled with iTRAQ labeling is susceptible to other reported peptide ions, whereas the sample matrix is complex (Minogue et al., 2015). Data-independent acquisition (DIA) is a focused quantitative method for protein mass spectrometry and can be applied to various quantitative proteomics experiments because of its accuracy and high efficiency (Sanda and Goldman, 2016). However, the DIA technique has not been used to screen adhesion/invasion related factors in *C. sakazakii*.

This study is the first time to investigate proteomic profiles of *C. sakazakii* using LC-MS/MS coupled with DIA labeling. The objective of this study is to investigate potential adhesion/invasion related factors as a useful reference for elucidation of the mechanism underlying adhesion to and invasion of the host cells by *C. sakazakii*. At the same time, the results presented herein will promote the understanding of the epidemiology of *C. sakazakii*.

## Materials and Methods

### Bacterial Strains and Sequence Typing

Based on the previous results from the adhesion/invasion assay, we selected 34 strains for sequence typing (Du et al., 2016). Based on the manufacturer’s instruction for the DNA Extraction Kit (Omega Bio-Tek, Norcross, GA, United States), genomic DNA from the 34 strains was obtained after overnight cultivation. Primers for seven housekeeping genes (*atpD*, *fusA*, *glnS*, *gltB*, *gyrB*, *infB*, and *pps*) were used for PCR (Baldwin et al., 2009), and the amplified products were sequenced with the Sanger chain termination method by Suzhou Jinweizhi Biotechnology Co., Ltd. (Suzhou, China). The sequencing results of the seven housekeeping genes were concatenated in a certain order to match with the PubMLST database and used to construct a phylogenetic tree by MEGA 6.0. The relationships among the 34 strains were analyzed via the neighbor-joining statistical method coupled with Tamura’s 3-parameter model (Joseph and Forsythe, 2012).

### Bacterial Cell Preparation for Proteomic Analysis and Protein Extraction

Based on the sequence typing results, SAKA80220 and SAKA80221, which showed the same sequence type (ST) but distinct adhesion/invasion capabilities, were selected for proteomic analysis (Ren et al., 2016; Han et al., 2018). These strains, which were stored in LB with 80% glycerol at −80°C, were incubated on LB agar plates for 16 h at 37°C. A single colony was picked and incubated in 10 mL of LB broth at 37°C for 16 h with shaking at 180 rpm. Overnight cultures were transferred to fresh culture medium (diluted 1:100) and grew until the OD_600_ reached 0.4−0.6. The *C. sakazakii* cells were then harvested by centrifugation at 5,000 × *g* for 5 min and washed twice with sterile PBS and once with sterile water. After centrifugation at 5,000 × g for 2 min, the bacterial cells were stored at −80°C until use.

A modified phenol-based protein extraction method was used (Wu et al., 2014). Cell pellets were mixed with 6 mL of protein extraction buffer [500 mM Tris-HCl buffer (pH 8.0), 50 mM EDTA, 700 mM sucrose, 100 mM KCl, 2%β-mercaptoethanol, 1 mM phenylmethylsulfonyl fluoride, and HCl pH 8.0] and ground for 10 min using a precooled mortar on ice. An equal volume of saturated phenol was added, and the samples were thoroughly ground for another 10 min on ice. For better dissolution, the protein solution was ultrasonicated for 5 min at 4°C before centrifugation at 5,500 × *g* for 10 min. The protein-containing phenol solution was removed into a new ice-cold centrifuge tube, mixed with 6 mL of extraction buffer and centrifuged at 5,000 × *g* for 10 min at 4°C. The phenol phase was transferred to a new centrifuge tube with ice-cold acetone and stored at −20°C overnight. Next, the samples were centrifuged at 5,000 × *g* for 10 min at 4°C, and then, the protein pellets were cleaned twice with 2 mL of ice-cold acetone before centrifugation at 5,000 × *g* for 5 min at 4°C. Finally, the protein concentration was quantified using the Bradford method (Bradford, 1976).

### Protein Digestion and Peptide Fractionation

A total of 100 μg of protein from each sample was digested using the FASP method (Wisniewski et al., 2009). Disulfide bonds were cleaved and the sites were blocked using 10 mM dithiothreitol (DTT) and 50 mM iodoacetamide (IAA), respectively. The proteins were then transferred to a 10-kDa filter and cleaned sequentially using 8 M urea and 50 mM NH_4_HCO_3_ at 12,000 × *g* and 20°C. Trypsin was added to each sample at a ratio of 1:50 (mass/mass), and the proteins were digested in 50 mM NH_4_HCO_3_ at 37°C for 16 h. A mixed sample was prepared with equal amount of digested peptides from each sample and separated into three fractions using a modified high-pH reversed-phase (High-pH RP) method (Dimayacyac-Esleta et al., 2015). Briefly, a homemade C18 stage tip was cleaned using 80% ACN/H_2_O after activation with methanol. Then, the stage tip was equilibrated with ammonium hydroxide (pH 10) before peptide loading. A series of ACN/ammonium hydroxide (pH 10) buffers – 6, 9, 12, 15, 18, 21, 25, 30, 35, and 50% – were utilized to elute the peptides.

### Mass Spectrometry and Data Analysis

#### Data-Dependent Acquisition Sample Acquisition and Spectral Library Generation

For generation of the spectral library, peptides from each eluted sample were mixed acquired three times with data dependent acquisition mode using Q Exactive HF (Bremen, Thermo Fisher). The peptide mixtures were separated on an EasyNano LC1000 system (Thermo, San Jose, CA, United States) using a homemade C18 column (3 μm, 75 μm × 15 cm) at a flow rate of 300 nL/min. A 120-min linear gradient was set as follows: 5% B (0.1% FA in 80% acetonitrile/H_2_O)/95% A (0.1% FA in H_2_O) to 8% B in 10 min, 8% B to 25% B in 83 min, 25% B to 45% B in 21 min, 45% B to 95% B in 1 min, and maintaining at 95% B for 5 min. For data acquisition, a top 20 scan mode with an MS1 scan range of 400–1,200 m/z was employed, and the other parameters were set as follows: the MS1 and MS2 resolutions were set to 120 and 30 K, respectively; automatic gain control (AGC) target for MS1 and MS2 were set to 3e6 and 1e6, respectively; the isolation window was 2.0 Th; the normalized collision energy was 27 eV; and the dynamic exclusion time was 20 s (Lin et al., 2018).

Data-dependent acquisition (DDA) raw files were searched against the UniProt protein database for *C. sakazakii* using Proteome Discoverer 2.1 (Thermo, San Jose, CA, United States). The protein sequence was affixed with the iRT fusion protein sequence (Biognosys). The parameter of search engine SequestHT was set as the following: digestion: trypsin; miss cleavages: 2; variable modifications: oxidation (M), deamidated (N, Q); fixed modifications: carbamidomethyl (C); peptide mass tolerance: ± 10 ppm; fragment mass tolerance: ± 0.02 Da; peptide FDR: less than 1%; protein FDR: q value less than 1%. The search results were transferred into a spectral library using Spectronaut 10 (Biognosys, Schlieren, Switzerland). Only high-confidence peptides were used for the generation of the spectral library. Fragment ions within a mass range of 300–1,800 m/z was retained, and peptides with less than three fragment ions were removed.

#### Data-Independent Acquisition Sample Acquisition and Data Analysis

Each sample treated with the same amount of iRT was analyzed by the DIA method (Muntel et al., 2015). For DIA acquisition, the procedure consisted of one full MS1 scan with a resolution of 60 K using an AGC of 3e6 and a maximum injection time of 20 ms; MS2 scan with a resolution of 30 K using an AGC of 1e6 and a maximum injection time of 45 ms. All the LC conditions were exactly the same as those used for DDA, which are listed above. DIA raw data was processed using Spectronaut 10; the default, which includes: peak detection; dynamic iRT; correction factor 1; interference correction and cross run normalization, enabled; and all peptides were filtered using *Q* value ≤ 0.01, was used for protein identification and quantitation. Average quantity of fragment ion areas from top three peptides was employed to compare protein abundance between samples. Screening of significantly differentially expressed proteins was performed with abundance ratios ≥3 or ≤0.33, and student test *P*-value ≤0.05.

### Gene Expression Analysis by Real-Time Quantitative PCR

To further validate the different expression patterns of the proteins, we selected five proteins that exhibited much higher expression levels in the strong-adhesive/invasive strain SAKA 80220 and six specific proteins that were only expressed in the strong-adhesive/invasive strain SAKA 80220. Eleven pairs of primers specific for target genes and one pair of primers specific for the *16S rRNA* gene, which was used as an internal reference, were designed and synthesized (Table 2). The total RNA was extracted from the strong-adhesive/invasive strain SAKA80220 and weak-adhesive/invasive strain SAKA80221, which were cultured to the logarithmic phase, by utilizing the EZNA^TM^ Bacterial RNA Kit (Omega Bio-Tek, Norcross, GA, United States). The RNA was reverse-transcribed to get the cDNA using the PrimeScript^TM^ RT Reagent Kit (TaKaRa Bio Inc.). The cDNA was further used as a template to perform qRT-PCR using a RealPlex 4 Master Cycler (Eppendorf). The mRNA expression levels of the genes were calculated according to the 2^– ΔΔCT^ method (Du et al., 2018).

## Results

### Sequence Typing of 34 Strains of *C. sakazakii*

**FIGURE 1:**
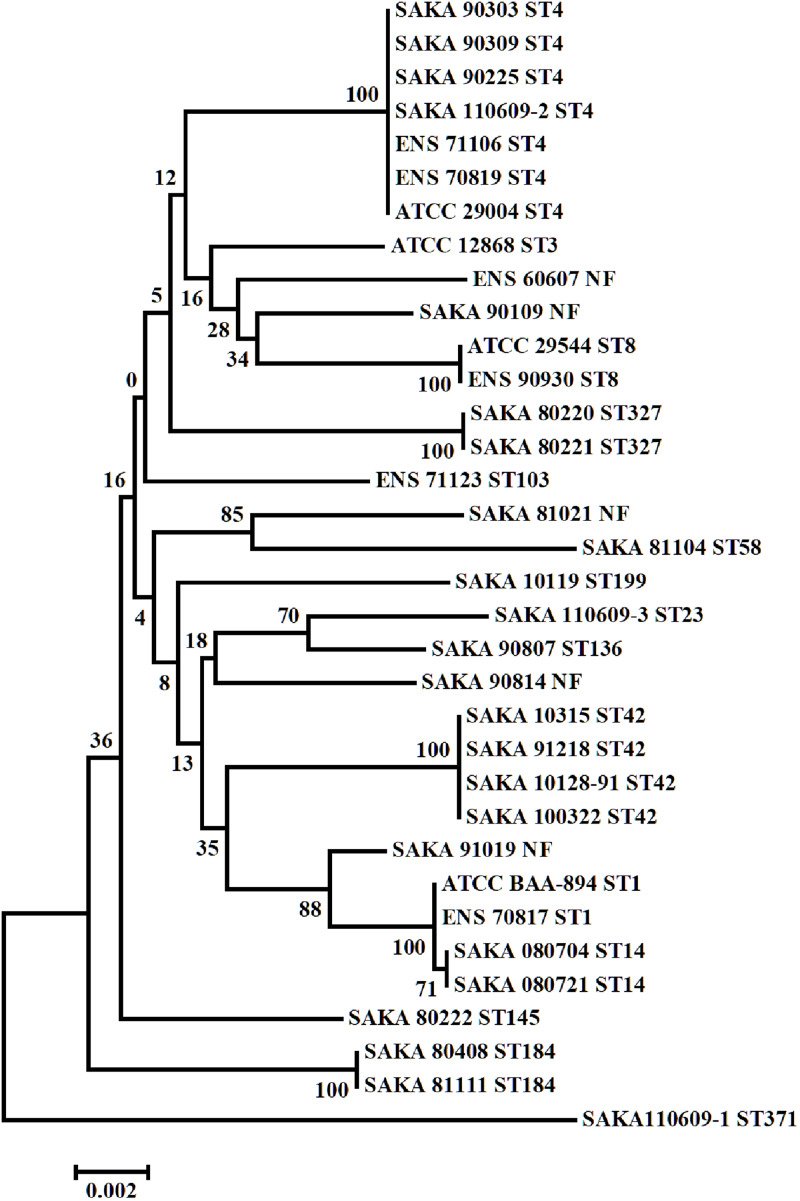
Phylogenetic tree of the 34 *C. sakazakii* strains.

Multilocus sequence typing (MLST) result indicated that the 34 *C. sakazakii* strains could be divided into 16 STs. The origin/source and the sequence types of 34 *C. sakazakii* strains were listed in Table 1, and the phylogenetic tree of the 34 *C. sakazakii* strains is shown in Figure 1.

**TABLE 1:** The origin/source and the sequence types of 34 *C. sakazakii* strains.

No.	Strain	Origin/source	Sequence type
1	ATCC BAA-894	ATCC/United States	1
2	ENS 70817	Consultation sample/China^a^	1
3	ATCC 12868	ATCC/United States	3
4	ATCC 29004	ATCC/United States	4
5	ENS 70819	Lactalbumin powder/United States	4
6	ENS 71106	Powdered infant formula/New Zealand	4
7	SAKA 110609-2	China^b^	4
8	SAKA 90225	Starbucks cake powder/United States	4
9	SAKA 90303	Whey powder/New Zealand	4
10	SAKA 90309	Lactalbumin powder/Netherlands	4
11	ENS 90930	China^c^	8
12	ATCC 29544	ATCC/United States	8
13	SAKA 080704	Whey powder/Netherlands	14
14	SAKA 080721	Skimmed milk powder/United States	14
15	SAKA 110609-3	China^b^	23
16	SAKA 100322	Whole milk/New Zealand	42
17	SAKA 91218	Whole milk powder/New Zealand	42
18	SAKA 10315	Whole milk/New Zealand	42
19	SAKA 10128-91	Pighead/Spain	42
20	SAKA 81104	Whole milk powder/Australia	58
21	ENS 71123	Skimmed milk powder/Canada	103
22	SAKA 90807	New Zealand	136
23	SAKA 80222	Sweet whey powder/France	145
24	SAKA 80408	Whole milk powder/Australia	184
25	SAKA 81111	Lactalbumin powder/United States	184
26	SAKA 10119	Whole milk/New Zealand	199
27	SAKA 80220	Infant growth formula/Netherlands	327
28	SAKA 80221	Whey powder/Austria	327
29	SAKA 110609-1	China^b^	371
30	SAKA 90109	Whey protein concentrate powder/United States	NF^d^
31	SAKA 90814	Austria	NF
32	ENS 60607	Ireland	NF
33	SAKA 91019	Milk powder/Austria	NF
34	SAKA 81021	Whey powder/New Zealand	NF

*^*a*^Isolates from consultation sample, Qinhuangdao Bureau, China. ^*b*^Isolates from school, Tianjin University of Science and Technology, China. ^*c*^Isolates from Jilin Bureau, China. ^*d*^NF means not found.*

**TABLE 2:** The primers and products for the qRT-PCR of the differentially expressed genes in SAKA80220 and SAKA80221.

Locus tag	Gene	Primer sequence (5′ → 3′)	Product size (bp)
ESA_02265	*flgk*	TGGCACCACCAACAATATCTCC	260
		TCAGACGGGTGATCTGTTCGTT	
ESA_04107	*waac*	GCGGTTTGTGGTGTCGGTA	201
		AGCCAGGGACGCAGTTGTT	
ESA_03599	*lptA*	TTGAAATCACCAACAGGCTCG	155
		GGCGGAATCCAGCATCAGTAG	
ESA_00581	*luxs*	ATCATCTGAACGGCAACGGT	219
		CCTCTTCCAGCGAGTGCATCT	
ESA_01966	*fumc*	CAGGAGTTCCCGCTTGCTAT	155
		TGGCTCTTGTTCACGTCATCG	
ESA_01988		AAGCCACCGCCCACAATC	248
		ATCCTGGCCTGAAGGCTGTAC	
ESA_01989		TGGCATATCCCGGTGAGTTT	178
		GACGTCCTTTGTTCGTGGTAGAG	
ESA_01990		GCTCGTTGGTGCGGATTTC	102
		TCGCTGATGCGGGTTTCTAT	
ESA_01856		TTCTTCCTGTTGTCGCCTGTG	164
		GTAGTCGTTGTTGCTGCCTTCA	
ESA_03837		AGCAGCAAATACGTGAAGAAGAGG	285
		CGCCACAAGCCTGAGCATAA	
ESA_00920	*soxS*	AACGCTGACCGACTGGATTG	279
		CCAGGCGACATCCGATACTTAC	
ESA_00047	*16S rRNA*	TTACGACCAGGGCTACACACG	102
		CGGACTACGACGCACTTTATGAG	

Among the 16 STs, ST4 included seven strains (7/34) was the dominant type, and ST42 included four strains (4/34), while ST1, ST8, ST14, ST184, and ST327 included two strains (2/34), respectively. Among the remaining strains, eight strains belonged to eight different ST types, and five strains were not found to match the sequence types in the PubMLST database. A strong-adhesive/invasive strain (SAKA80220) and a weak-adhesive/invasive strain (SAKA80221), both belonging to the ST327 type, were selected for proteome analysis using DIA techniques.

### Database Construction and Protein Identification

Quantitative DIA analysis was performed on two groups of bacterial samples, and a total of 2,319 proteins were identified for three biological repeats, 2,203 proteins among which were reliably quantified. The result of the correlation analysis between the samples showed that the sample correlation within the group is very high and it close to 1, suggesting a good repeatability of the experiment (Supplementary Figure S1). The results of the cluster analysis indicated that intra-group repeatedly clustered together, suggesting a higher intra-group repeatability (Supplementary Figure S2). Screening of differentially expressed proteins was performed with abundance ratios ≥3 or ≤0.33, and *P* values ≤0.05. A total of 210 differentially expressed proteins were identified. Of these differentially expressed proteins, 67 were highly expressed in SAKA80220 (strong-adhesive/invasive strain) compared to SAKA80221 (weak-adhesive/invasive strain, ≥3-fold at *p* ≤ 0.05) and the expression level of 143 proteins were lower in SAKA80220 compared to SAKA80221 (≤0.33-fold at *p* ≤ 0.05) (Supplementary Table S1). Some of proteins, such as FlgK, WaaC, LptA, LuxS, Fumc, KatG, and YtfE could be considered to be potential factors related to the adhesion and invasion processes. Notably, there were six specific genes encoding electron transport complex proteins (*rnfC*, *rnfD*, and *rnfG*), transcriptional regulators (*soxS* and ESA_03837), and hypothetical protein (ESA_01856) in the strong-adhesive/invasive strain SAKA80220 (Table 3).

**TABLE 3:** The putative adhesion/invasion-related proteins and hypothetical proteins in the strong-adhesive/invasive strain SAKA80220.

	Gene	Uniprot accession	Fold change	*P* value	KEGG annotation	STRING annotation
Lipopolysaccharide biosynthesis	*waaC*	A7MQ89	3.31	0.0006	eas × 00540 Lipopolysaccharide biosynthesis	ADP-heptose
	*lptA*	A7MJE3	3.02	0.02102	0	Lipopolysaccharide transport periplasmic protein
Flagella	*flgk*	A7MFQ0	8.95	0.00629	esa02040 × Flanellar assemly	Flagellar hook-associated protein FlgK
Iron-sulfur cluster repair protein	*ytfE*	A7MM70	3.38	0.0059	0	Iron-sulfur cluster repair protein YtfE
Microbial metabolism in diverse environments	*luxS*	A7MJ28	3.55	0.00582	eas × 02024 Quorum sensing	S-ribosylhomocysteinase
Metabolic pathways	*hemA*	A7MKC0	3.31	0.01866	esa01110 Biosynthesis of secondary metabolites esa01100 Metabolic pathways	Glutamyl-tRNA reductase
	*mtnD*	A7MK12	4.38	0.0047	eas × 00270 Cysteine methionine metabolism	Hypothetical protein
	*fumC*	A7MMN0	3.68	0.00301	esa00020 × Citrate cycle; eas × 00620 Pyruvate metabolism	Fumarate hydratase
	*lacZ*	A7MN76	20.95	0.00262	eas × 00511 other glycan degradation; eas00600 × Sphingolipid metabolism	beta-D-galactosidase
	*katG*	A7MJS4	6.41	0.00057	esa00360 × Phenylalanine metabolism; esa00380 × Tryptophan metabolism	Catalase-peroxidase
Other proteins	*rnfC*	A7MMK9	∞	0.0004	0	NADH:ubiquinone oxidoreductase, subunit RnfC
	*rnfD*	A7MML0	∞	0.0066	0	NADH:ubiquinone oxidoreductase, subunit RnfD
	*rnfG*	A7MML1	∞	0.00038	0	NADH:ubiquinone oxidoreductase, subunit RnfG
	ESA_01856	A7MK79	∞	0.0202	0	Hypothetical protein
	ESA_03837	A7ML73	∞	0,0112	0	Xenobiotic response element family of transcriptional regulator
	*soxS*	A7MH41	∞	9.51426E-05	0	DNA-binding transcriptional regulator

*“∞” Indicates that the proteins are only detected in the strong-adhesive/invasive strain.*

### Functional Classification of High Expression Differential Proteins in the Strong-Adhesive/Invasive Strain SAKA80220

**FIGURE 2:**
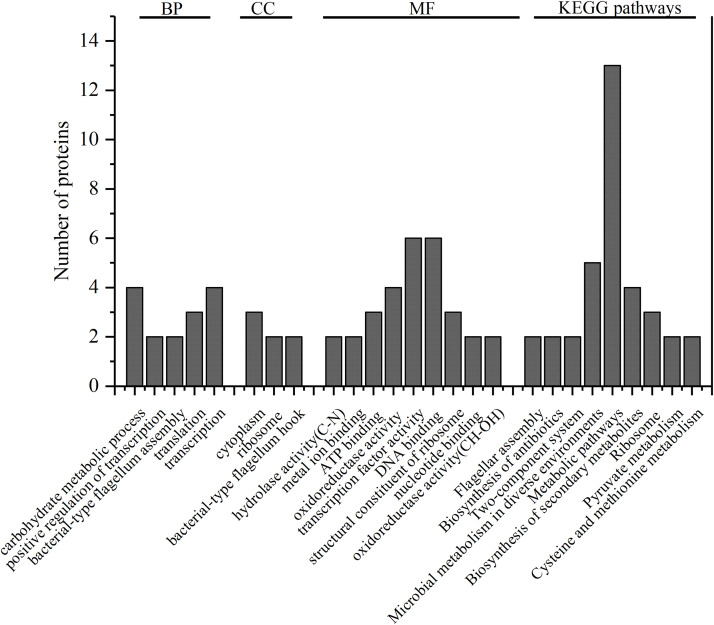
GO annotation of 48 identified proteins. The number of the proteins which significantly enriched categories in biological process (BP), cell component (CC), and molecular function (MF) are shown on the *y*-axes; KEGG pathways of 47 identified proteins, the number of the proteins in the nine most significantly enriched pathways are shown on the *y*-axes.

Among the 67 high expression differential proteins in the strong-adhesive/invasive strain SAKA80220, 48 proteins belonged to specific genomic information categories in UniProt^1^, and the other 19 were hypothetical or unknown proteins that could not be identified in this database. The summarized GO annotation data of the 48 proteins is shown in Figure 2.

GO annotation showed that these 48 proteins were mainly enriched in the following function categories. In biological processes (BP), 29 proteins were mainly involved in carbohydrate metabolic process, positive regulation of transcription, bacterial-type flagellum assembly, translation and transcription. In the cell component (CC) category, 18 proteins were mostly associated with the cytoplasm, ribosome and bacterial-type flagellum hook. In the molecular function (MF) category, 38 proteins were mainly distributed in hydrolase activity (C–N), metal ion binding, ATP binding, oxidoreductase activity, transcription factor activity, DNA binding, structural constituent of ribosome, nucleotide binding and oxidoreductase activity (CH–OH). Notably, according to GO annotation, a single protein may play more than one role.

### KEGG Analysis of Differentially Expressed Proteins

Kyoto Encyclopedia of Genes and Genomes^2^ (KEGG, http://www.kegg.jp/kegg/tool/map_pathway2.html) analysis provided additional information regarding the enrichment of expressed proteins in each pathway furthermore. In this analysis, 47 out of the 67 proteins were annotated to 31 pathways, and the top 9nine pathways were significantly enriched (Figure 2), namely, flagellar assembly, biosynthesis of antibiotics, two-component system, microbial metabolism in diverse environments, metabolic pathways, biosynthesis of secondary metabolites, ribosome, pyruvate metabolism, and cysteine and methionine metabolism. There was a strong correlation between the enriched proteins in KEGG pathways and the enrichment of proteins in the BP, CC, and MF categories. Furthermore, the detailed analysis of the highly expressed differential proteins and the specific proteins in strong-adhesive/invasive strain SAKA80220 were performed using the protein-protein interaction network analysis database^3^ (STRING, http://string-db.org).

### STRING Analysis of Distinct Proteins

**FIGURE 3:**
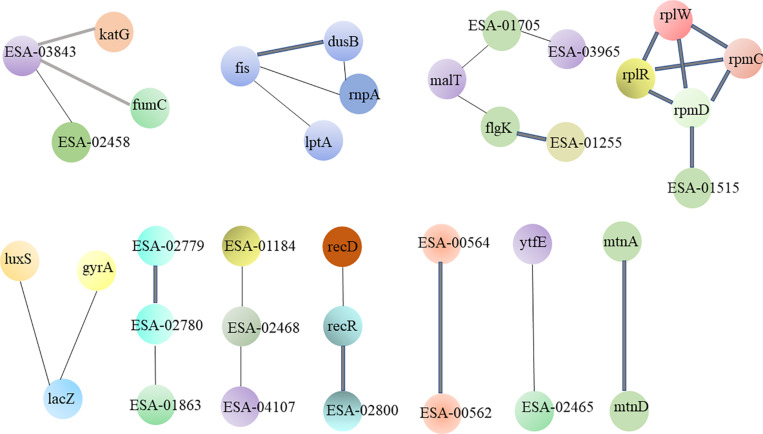
The interacted network of protein-protein was analyzed by STRING. These differential proteins which are high expression in strong-adhesive/invasive strain SAKA80220 compared to weak-adhesive/invasive strain SAKA80221 are mainly concentrated in: flagellar proteins; 50s ribosomal subunit protein; LPS biosynthesis proteins and hypothetical proteins.

The STRING database was used to comprehensively investigate the interactions among the various proteins. The detailed protein interactions are shown in Figure 3. The line width is positively correlated with the change extent. The following proteins exhibited high numbers of interactions: flagellar proteins; 50s ribosomal subunit protein; LPS biosynthesis proteins; proteins associated with microbial metabolism in diverse environments; proteins associated with nucleotide metabolism and DNA replication; and hypothetical proteins.

### Validation of Gene Expression by Quantitative Real-Time qPCR

**FIGURE 4:**
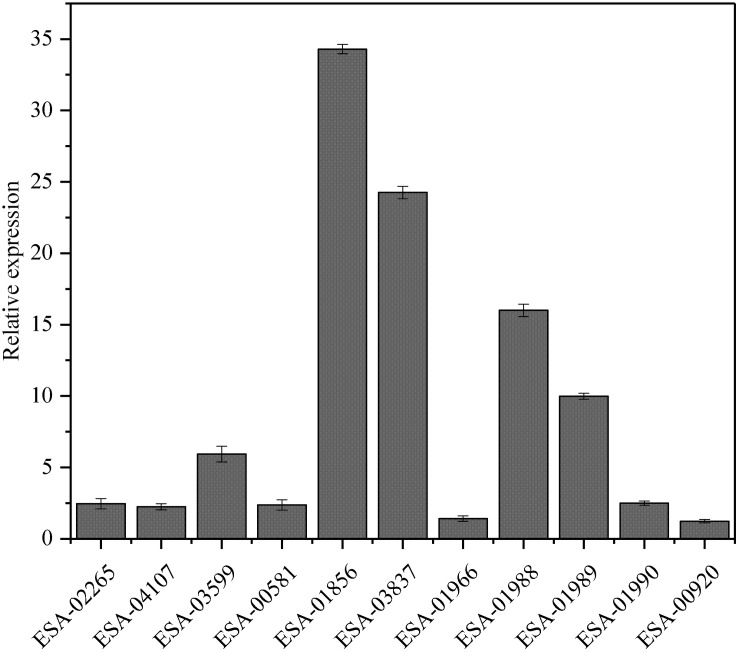
Real-time qPCR analysis of transcription levels of 11 genes in the two strains.

To investigate the relationships between protein expression and the corresponding mRNA expression patterns, real-time qPCR was used to analyze the dynamic transcriptional expression of 11 representative high expression proteins with *16S rRNA* as the reference gene. The results demonstrated that a majority of the genes exhibited coincident protein and mRNA expression patterns, except for the gene of *fumc*(ESA_01966)and *soxS* (ESA_00920), which did not show changes in expression between the strains with distinct adhesion/invasion capabilities (Figure 4).

## Discussion

Based on our previous results (Du et al., 2016) from adhesion/invasion analysis coupled with sequence typing, we selected the strong-adhesive/invasive strain SAKA80220 and the weak-adhesive/invasive strain SAKA80221 as strains that exhibited distinct adhesion/invasion capabilities but were close in evolutionary relationship for comparative proteomics analysis. By LC-MS/MS coupled with DIA, a total of 2,203 proteins were identified, among which, 67 proteins were high expression in the strong-adhesive/invasive strain SAKA80220 compared to the weak-adhesive/invasive strain SAKA80221 (≥3-fold at *p* ≤ 0.05). According to the STRING results, some proteins with unknown functions and proteins associated with the flagellar assembly, lipopolysaccharide synthesis, LuxS/AI-2, energy metabolic pathways, iron-sulfur cluster exhibited distinct biosynthesis in the two strains.

The flagellum is the primary organ associated with bacterial motility and is composed of three main parts: basal body, hook, and filament. The role of the flagellum hook is to connect the basal body and filament (Morimoto and Minamino, 2014). The flagellar hook-associated protein FlgK, encoded by the *flgK* (ESA_02265) gene, is regarded as an important virulence factor for invasion in other microorganisms, such as *Escherichia coli* and *Salmonella enterica* (Gauger et al., 2007; Shah et al., 2011). In addition, some studies have shown that mutation of *flgK* leads to reduced motility and virulence-related enzyme activity in bacteria, in turn leading to decreased pathogenicity (Pallen et al., 2005; Terashima et al., 2017). In this study, the expression of the FlgK protein in the strong-adhesive/invasive strain SAKA80220 was 8.95-fold higher than that in the weak-adhesive/invasive strain SAKA80221, which is consistent with the mRNA levels observed by real-time qPCR. Taken together, all the studies indicate that the *flgK* gene is responsible for improvement of flagellar motility, thereby enhancing the ability of bacteria to adhere to and invasion of the host cells.

Lipopolysaccharides are the main functional and structural components of the cell surfaces of Gram-negative bacteria and have also been shown to be the main virulence factors (Raetz and Whitfield, 2002). LPSs are composed of lipid A, core oligosaccharides and O-antigens. Lipid A represents the hydrophobic component of LPSs located in the outer leaflet of the outer membrane, while core polysaccharides and O-antigen repeats are displayed on the surfaces of bacterial cells (Wang and Quinn, 2010). In our study, two proteins were differently expressed in the strong-adhesive/invasive strain and the weak-adhesive/invasive strain which were related to LPS biosynthesis and translocation, including *waaC* (ESA_04107) and *lptA* (ESA_03599). *WaaC* encodes heptose-transferase I, as a key gene in the lipopolysaccharide biosynthesis pathway. In *E. coli*, the heptose-transferase I WaaC transfers the first core heptose to Kdo2-lipid A, which is the main active component of endotoxin (Moreau et al., 2008). In this study, WaaC was expressed much higher levels in the strong-adhesive/invasive strains than in the weak-adhesive/invasive strains (3.31-fold). And the high expression of WaaC may increase LPS synthesis and thereby enhance the ability of bacteria to adhere to and invasion of the host cells. In Gram-negative bacteria, LPS is biosynthesized in the cytoplasm, and then, translocated to the periplasmic side of the inner membrane. During translocation, the periplasmic LPS transport protein LptA forms a bridge between the inner membrane and the outer membrane via interactions with the other LPS transport proteins LptB–LptG, thereby facilitating the transfer of LPS from the inner membrane to the outer membrane. It was also reported that LptA may act as a periplasmic chaperone for LPS transport across the periplasm because LptA plays a role in periplasmic localization and has been shown to bind both LPS and lipid A *in vitro* (Sperandeo et al., 2007, 2011; Tran et al., 2008). In this study, the expression of the LptA protein in the strong-adhesive/invasive strain was 3.02 times that in the weak-adhesive/invasive strain, indicating the involvement of this protein in pathogenesis. Our results proved that the biosynthesis and translocation of LPS are closely associated with the adhesion/invasion capability of *C. sakazakii*.

*S*-ribosylhomocysteinase (LuxS, ESA_00581) is involved in the synthesis of the signal molecule autoinducer 2 (AI-2), which is secreted by bacteria and used to respond to both cell density and metabolism (Ye et al., 2016a, b). Many studies have indicated the importance of LuxS/AI-2 in BP of Gram-negative and Gram-positive bacteria, including antibiotic production, biofilm formation, and carbohydrate metabolism (Ma et al., 2017). Notably, the AI-2 plays an important role in controlling virulence factors during early growth of *Edwardsiella* (Han et al., 2010). Compared to the wild-type, the luxS-deleted mutants of *E. coli* SE15 showed dramatically decreased biofilm formation (Kang et al., 2016). The *Actinobacillus pleuropneumoniae* luxS mutant showed reduced adhesion ability, at the same time, AI-2 could increase adhesion and biofilm formation of *A. pleuropneumoniae* independent of LuxS (Li et al., 2011). In this study, the expression of LuxS protein in the strong-adhesive/invasive strain was 3.55 times higher than that in the weak-adhesive/invasive strain, which is consistent with the observed mRNA levels. According to accumulating experimental evidence regarding the role of LuxS/AI-2, LuxS/AI-2-mediated quorum sensing may play crucial roles in the formation of biofilms and generation of adhesion/invasion related factors in *C. sakazakii.*

Another differentially expressed protein which was found is the catalase-peroxidase KatG. Catalase-peroxidase is widely found in animals, plants and microorganisms, such as animal livers and cells, organelles of plants, as well as bacteria. The biological functions of catalase-peroxidase mainly include decomposition of hydrogen peroxide, inhibition of cellular oxidation stimulation and cell senescence (Song et al., 2005). In this study, the expression of the KatG protein in the strong-adhesive/invasive strain SAKA80220 was 6.41-fold higher than that in the weak-adhesive/invasive strain SAKA80221. In the KEGG pathway, KatG was predicted as a catalase-peroxidase involved in the tryptophan metabolism and phenylalanine metabolism of *C. sakazakii*. By comparing the protein sequence of KatG in *C. sakazakii* and *E. coli*, the similarity between the two proteins was high (84.55%). It’s reported that the expression of catalase-peroxidase KatG in *E. coli* can increase its tolerance to oxidative stress (Kang et al., 2011). By comparing the protein sequence of KatG in *C. sakazakii* and *Mycobacterium tuberculosis*, we found that the similarity between the two proteins was 57.71%. The isoniazid resistance of *Mycobacterium tuberculosis* is closely related to the deletion and mutation of *katG* gene. When the *katG* locus is mutated, the isoniazid cannot be activated and converted into an effective bactericidal form. These mutant strains showed reduced virulence when challenged mice, and the introduction of exogenous *katG* gene into these strains can restore virulence (Ng et al., 2004). Therefore, the high expression of KatG protein may be associated with the high adhesion/invasion capability of *C. sakazakii*.

To obtain sufficient nutrients and energy from host cells, bacteria must develop multitudinous enzymes and metabolic pathways. Therefore, the demand for nutrients is considered to be the driving factor for the evolution of virulence (Gorke and Stulke, 2008; Eisenreich et al., 2010). Interestingly, proteomics results have indicated that the expression of the β-galactosidase LacZ in the strong-adhesive/invasive strain was 20.95-fold higher than that in the weak-adhesive/invasive strain. It is well known that cells activate the expression of the lactose operon when glucose levels are very low in the metabolic pathway of *E. coli*, and then, the *E. coli* cells allow the β-galactosidase to catabolize lactose to galactose and glucose to meet metabolic energy requirements. The *lacZ* gene was expressed at significantly high levels in the strong-adhesive/invasive strain, perhaps due to initiation of the lactose metabolic pathway to meet energy needs to maintain cellular activity in the adverse environment of the host cells. Fumarase is an important enzyme in the tricarboxylic acid (TCA) cycle, catalyzing the reversible hydration/dehydration of fumarate to L-malate (Shimoyama et al., 2007). Several studies have indicated that *E. coli* seemed to utilize highly responsive regulatory elements to adapt the levels of the fumarase enzymes (*fumA*, *fumB*, and *fumC*) under different cell growth conditions for energy generation and that the *fumC* (ESA_01966) gene is affected by the type of carbon source used for cell growth (Park and Gunsalus, 1995; Tseng et al., 2001). In this study, the expression of fumarate hydratase (*fumC*) in the strong-adhesive/invasive strain was 3.68 times higher than that in the weak-adhesive/invasive strain. The FumC protein may be associated with adverse environments and virulence during pathogenic infection.

In this experiment, the expression level of iron–sulfur cluster repair protein YtfE in the strong-adhesive/invasive strain SAKA80220 was 3.38 times that of the weak-adhesive/invasive strain SAKA80221. YtfE is an important diiron protein and contains a non-heme dual-core iron center, which has a bridging ligand linking μ-oxo and μ-carboxylate and six histidine residues coordinated to iron ions. One role of YtfE is to reverse the nitrosation damage by directly or indirectly releasing nitric oxide (NO) from the nitrosated protein into the cytoplasm in *E. coli* (Balasiny et al., 2018). As we all known, NO is a key signaling and defense molecule in biological systems (Corker and Poole, 2003). Therefore, in this experiment, the high expression of YtfE protein may be benefit to improve the ability of *C. sakazakii* to adhere to host cells.

Furthermore, notably, six specific proteins are only expressed in the strong-adhesive/invasive strain SAKA80220, including three electron transport complex proteins. ESA_01988, ESA_01989, and ESA_01990, which act as part of the membrane complex involved in electron transport. The high expression of these proteins indicates that the metabolic activity of the strong-adhesive/invasive strain SAKA80220 was relatively high and the additional stored energy might be helpful for adhesion to or invasion of the host cells. In addition to the genes mentioned above, two genes encoding transcriptional regulators (*soxS* and ESA_03837) and a gene encoding hypothetical protein (ESA_01856) were also identified in the current study. Sequence alignment showed that these proteins are widely present in *Cronobacter* and share high identity (85−100%) among different *Cronobacter* species. However, ESA_01856 and ESA_03837 were not found to be similar in other bacteria. Therefore, analysis of these proteins with unknown functions would be helpful for comprehension of the basis of the pathogenicity of *Cronobacter*. Further studies are required to confirm the functions of the hypothetical proteins, as the differential expression demonstrates that some proteins may play vital roles in the adhesion to and invasion of the host cells by *C. sakazakii*.

In this study, MLST was performed to analyze 34 *C. sakazakii* strains and two strains with the same ST but with distinct adhesion/invasion capabilities were selected for identification of differentially expressed proteins using DIA proteomic analysis. A total of 210 proteins exhibited differential expression patterns with abundance ratios ≥3 or ≤0.33 and *P* values ≤0.05, 67 proteins were high expression in the strong-adhesive/invasive strain SAKA80220 compared with the weak-adhesive/invasive strain SAKA80221. These proteins, which may impact the adhesion/invasion capability, were mainly associated with the flagellar assembly, lipopolysaccharide synthesis, LuxS/AI-2, energy metabolic pathways and iron–sulfur cluster. Furthermore, the different expression proteins were further ascertained by real-time qPCR at the mRNA level. This research provided a useful reference for further studies on the pathogenic mechanism of *C. sakazakii*.

## Data Availability Statement

The mass spectrometry proteomics data have been deposited to the ProteomeXchange Consortium with the dataset identifier PXD018508. Other datasets generated for this study are included in the article/Supplementary Material.

## Author Contributions

XD and XW mainly contributed to the designing of the experiments, performing, and writing of the manuscript. PL, TD, XD, and SW devoted their efforts to conceive and design the work. XD and PL made a great contribution in designing and performing the experiments and modifying of the later manuscript. All authors agreed to be accountable for the content of the work and contributed to the conception of the study.

## Conflict of Interest

The authors declare that the research was conducted in the absence of any commercial or financial relationships that could be construed as a potential conflict of interest.

## Footnotes

1 https://david.ncifcrf.gov/tools.jsp

2 http://www.kegg.jp/kegg/tool/map_pathway2.html

3 http://string-db.org

## Supplementary Material

FIGURE S1 The correlation analysis between the protein samples of the strong-adhesive/invasive strain SAKA80220 and weak-adhesive/invasive strain SAKA80221.

FIGURE S2 The cluster analysis of the protein samples of the strong-adhesive/invasive strain SAKA80220 and weak-adhesive/invasive strain SAKA80221.

TABLE S1 The detailed information of the differentially expressed proteins in the two strains with distinct virulence.
