# Supplementary figures and images for: Comparative Proteomic Analysis of Adhesion/Invasion Related Proteins in Cronobacter sakazakii Based on Data-Independent Acquisition Coupled With LC-MS/MS
Source: Front Microbiol. 2020 Jun 9;11:1239. doi: 10.3389/fmicb.2020.01239 (PMC7296052; doi:10.3389/fmicb.2020.01239)

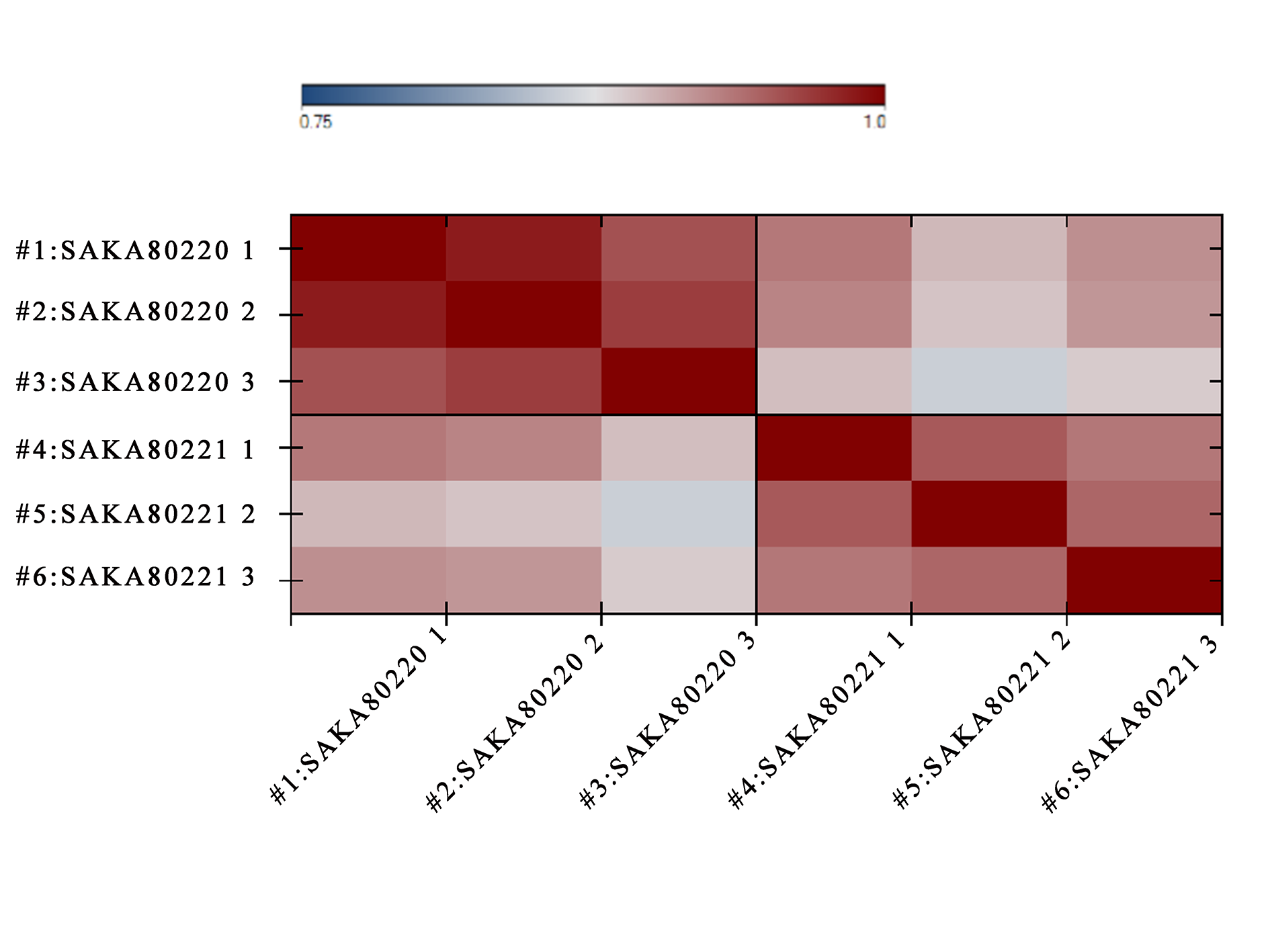

Supplement: FIGURE S1 — The correlation analysis between the protein samples of the strong-adhesive/invasive strain SAKA80220 and weak-adhesive/invasive strain SAKA80221. [file Image_1.TIF]

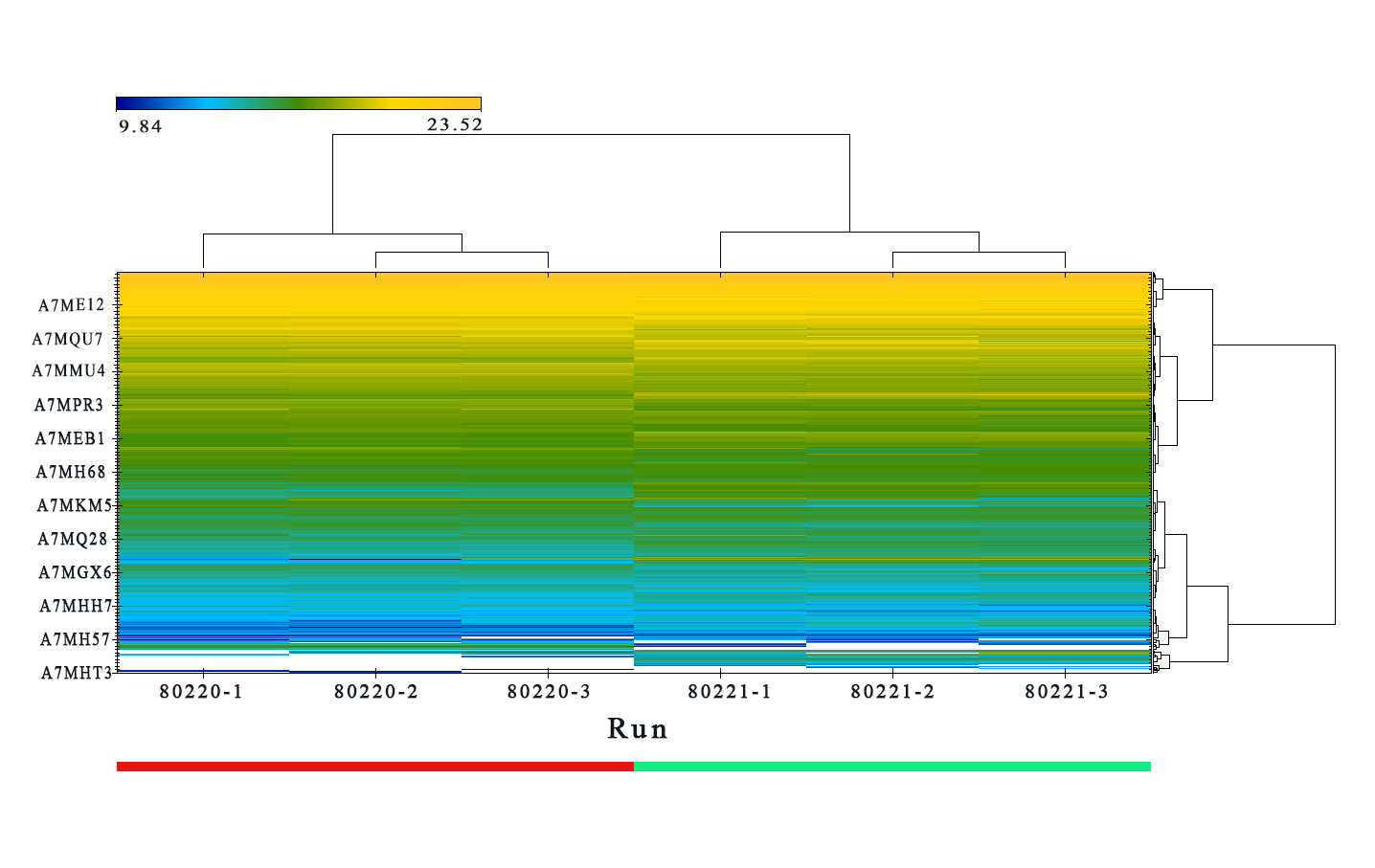

Supplement: FIGURE S2 — The cluster analysis of the protein samples of the strong-adhesive/invasive strain SAKA80220 and weak-adhesive/invasive strain SAKA80221. [file Image_2.TIF]
