# Supplementary material for: Comparative Proteomic Analysis of Adhesion/Invasion Related Proteins in Cronobacter sakazakii Based on Data-Independent Acquisition Coupled With LC-MS/MS
Source: Front Microbiol. 2020 Jun 9;11:1239. doi: 10.3389/fmicb.2020.01239 (PMC7296052; doi:10.3389/fmicb.2020.01239)
Supplement: TABLE S1 — The detailed information of the differentially expressed proteins in the two strains with distinct virulence. [file Table_1.docx]

Table S1

The detailed information of the differentially expressed proteins in the two strains with distinct adhesion/invasion capability.

| Gene | Uniprot accession | Protein Descriptions | Fold change | P Value |
| --- | --- | --- | --- | --- |
| *rnfC* | A7MMK9 | NADH:ubiquinone oxidoreductase, subunit RnfC | ∞ | 0.0004 |
| *rnfD* | A7MML0 | NADH:ubiquinone oxidoreductase, subunit RnfD | ∞ | 0.0066 |
| *rnfG* | A7MML1 | NADH:ubiquinone oxidoreductase, subunit RnfG | ∞ | 0.00038 |
| ESA-01856 | A7MK79 | Hypothetical protein | ∞ | 0.0202 |
| ESA-03837 | A7ML73 | Hypothetical protein | ∞ | 0,0112 |
| ESA-00920 | A7MH41 | Hypothetical protein | ∞ | 9.51426E-05 |
| ESA_00860 | A7MKV4 | Alpha-acetolactate decarboxylase | 22.0019 | 0.03815 |
| lacZ | A7MN76 | Beta-galactosidase | 20.9493 | 0.00262 |
| ESA_01551 | A7MMH3 | Uncharacterized protein | 15.1075 | 0.04097 |
| patA | A7MIU0 | Putrescine aminotransferase | 14.2691 | 0.00003 |
| ESA_01515 | A7MNB1 | Uncharacterized protein | 10.2064 | 0.00309 |
| ESA_02962 | A7MLR4 | Uncharacterized protein | 9.3429 | 0.01674 |
| epd | A7MJQ2 | D-erythrose-4-phosphate dehydrogenase | 9.8891 | 0.00053 |
| ESA_00730 | A7MGX0 | Uncharacterized protein | 9.2927 | 0.00000 |
| flgK | A7MFQ0 | Flagellar hook-associated protein 1 | 8.9535 | 0.00629 |
| ESA_02702 | A7MNQ1 | Uncharacterized protein | 8.9403 | 0.00002 |
| ESA_00562 | A7MJ47 | Uncharacterized protein | 8.7526 | 0.00003 |
| ESA_00836 | A7MKW0 | Uncharacterized protein | 8.0098 | 0.00082 |
| ESA_01863 | A7MK86 | Uncharacterized protein | 7.8067 | 0.00002 |
| ESA_02458 | A7MEQ9 | Uncharacterized protein | 7.6446 | 0.00016 |
| ESA_pESA3p05459 | A7MRQ1 | Uncharacterized protein | 7.4895 | 0.00019 |
| ESA_02502 | A7MF18 | Uncharacterized protein | 6.9528 | 0.00010 |
| ESA_03956 | A7MQ03 | Uncharacterized protein | 6.5661 | 0.00002 |
| katG | A7MJS4 | Catalase-peroxidase | 6.4051 | 0.00057 |
| ESA_00564 | A7MJ35 | Uncharacterized protein | 5.8580 | 0.00014 |
| rpmD | A7MPG8 | 50S ribosomal protein L30 | 5.5372 | 0.00003 |
| ESA_03988 | A7MMZ6 | Uncharacterized protein | 5.4225 | 0.00084 |
| ESA_02302 | A7MG22 | Uncharacterized protein | 4.9025 | 0.02124 |
| ESA_02535 | A7MEZ3 | Uncharacterized protein | 4.8253 | 0.01295 |
| ESA_02982 | A7MN81 | Uncharacterized protein | 4.6174 | 0.00018 |
| ESA_01705 | A7MLG4 | Uncharacterized protein | 4.5766 | 0.00155 |
| ESA_01702 | A7MLG1 | Uncharacterized protein | 4.4229 | 0.00629 |
| mtnD | A7MK12 | Acireductone dioxygenase | 4.3809 | 0.00521 |
| ESA_01255 | A7MJK8 | Uncharacterized protein | 4.2317 | 0.00260 |
| ESA_03712 | A7MQN8 | Uncharacterized protein | 4.2033 | 0.00463 |
| ESA_01962 | A7MMN4 | Uncharacterized protein | 4.1660 | 0.00496 |
| ESA_00947 | A7MHU1 | Uncharacterized protein | 4.0199 | 0.00009 |
| ESA_02583 | A7MIY4 | Uncharacterized protein | 3.9903 | 0.00401 |
| ESA_02574 | A7MIY9 | Uncharacterized protein | 3.8773 | 0.00769 |
| ESA_00601 | A7MHZ9 | Uncharacterized protein | 3.8289 | 0.00496 |
| ESA_01065 | A7MLL4 | Uncharacterized protein | 3.7281 | 0.00030 |
| ESA_02174 | A7MNU3 | UPF0229 protein ESA_02174 | 3.6995 | 0.00580 |
| fumC | A7MMN0 | Fumarate hydratase class II | 3.6774 | 0.00301 |
| rplW | A7MPI0 | 50S ribosomal protein L23 | 3.6643 | 0.00003 |
| ESA_00554 | A7MJ52 | Uncharacterized protein | 3.6357 | 0.00021 |
| ESA_00957 | A7MHT5 | Uncharacterized protein | 3.6148 | 0.00095 |
| ESA_03843 | A7MNF8 | Superoxide dismutase | 3.5707 | 0.00039 |
| fis | A7MJA9 | DNA-binding protein Fis | 3.5521 | 0.00328 |
| luxS | A7MJ28 | S-ribosylhomocysteine lyase | 3.5501 | 0.00045 |
| ESA_03498 | A7MIS7 | Uncharacterized protein | 3.4667 | 0.00006 |
| ESA_04390 | A7ME24 | Uncharacterized protein | 3.4023 | 0.00561 |
| ESA_04113 | A7MID4 | Uncharacterized protein | 3.3880 | 0.03540 |
| ytfE | A7MM70 | Iron-sulfur cluster repair protein | 3.3841 | 0.00329 |
| ESA_02966 | A7MLR7 | Uncharacterized protein | 3.3501 | 0.01395 |
| ESA_03399 | A7MG95 | Uncharacterized protein | 3.3157 | 0.00072 |
| ESA_03295 | A7MIA3 | Uncharacterized protein | 3.3148 | 0.00157 |
| ESA-04107 | A7MQ89 | Uncharacterized protein | 3.3101 | 0.0006 |
| hemA | A7MKC0 | Glutamyl-tRNA reductase | 3.3096 | 0.01866 |
| mutH | A7MR27 | DNA mismatch repair protein MutH | 3.2540 | 0.00548 |
| ESA_01584 | A7MMF0 | Uncharacterized protein | 3.2507 | 0.01047 |
| ESA_00026 | A7MPG9 | 50S ribosomal protein L15 | 3.2356 | 0.00760 |
| ESA_01922 | A7MPX7 | Uncharacterized protein | 3.2146 | 0.00382 |
| gyrA | A7MPB0 | DNA gyrase subunit A | 3.2060 | 0.01003 |
| ESA_01661 | A7MGG9 | Uncharacterized protein | 3.1734 | 0.01299 |
| ESA_00373 | A7MJT0 | Uncharacterized protein | 3.1300 | 0.00002 |
| rpmC | A7MPH1 | 50S ribosomal protein L29 | 3.0966 | 0.00582 |
| lptA | A7MJE3 | Lipopolysaccharide export system protein LptA | 3.0212 | 0.02102 |
| thiI | A7MFH3 | tRNA sulfurtransferase | 0.3322 | 0.00096 |
| ESA_00751 | A7MGU9 | Uncharacterized protein | 0.3319 | 0.00005 |
| ESA_00847 | A7MP47 | Nucleoside permease | 0.3314 | 0.02495 |
| aas | A7MR36 | Bifunctional protein Aas | 0.3300 | 0.00002 |
| ESA_03735 | A7MQK6 | Uncharacterized protein | 0.3278 | 0.00009 |
| ESA_00476 | A7MR25 | Uncharacterized protein | 0.3257 | 0.00095 |
| ESA_01345 | A7MEE2 | Uncharacterized protein | 0.3242 | 0.03353 |
| speA | A7MJR1 | Biosynthetic arginine decarboxylase | 0.3234 | 0.00004 |
| ppk | A7ML14 | Polyphosphate kinase | 0.3230 | 0.00266 |
| ESA_01181 | A7MHE6 | Uncharacterized protein | 0.3197 | 0.00059 |
| ESA_02439 | A7MET0 | Uncharacterized protein | 0.3195 | 0.00074 |
| ESA_00684 | A7MH07 | L-aspartate oxidase | 0.3191 | 0.00020 |
| ESA_01188 | A7MHD7 | Uncharacterized protein | 0.3189 | 0.00177 |
| ESA_02087 | A7MF56 | Uncharacterized protein | 0.3180 | 0.00201 |
| ESA_00617 | A7MHZ1 | Uncharacterized protein | 0.3179 | 0.00017 |
| ESA_01353 | A7MED2 | Protein phosphatase CheZ | 0.3163 | 0.00121 |
| ESA_02075 | A7MF78 | Uncharacterized protein | 0.3152 | 0.00633 |
| ESA_03361 | A7MIG1 | Zinc-type alcohol dehydrogenase-like protein | 0.3125 | 0.00452 |
| ESA_01828 | A7MPR6 | Uncharacterized protein | 0.3121 | 0.01693 |
| ESA_01291 | A7MP21 | Uncharacterized protein | 0.3108 | 0.01372 |
| ESA_00691 | A7MGZ9 | Signal peptidase I | 0.3086 | 0.01862 |
| ESA_01176 | A7MHF7 | Uncharacterized protein | 0.3085 | 0.00068 |
| ESA_00643 | A7MHW8 | Uncharacterized protein | 0.3084 | 0.00610 |
| ESA_00276 | A7MM13 | Uncharacterized protein | 0.3076 | 0.00856 |
| ESA_04329 | A7MGD8 | Ferrous iron transport protein B | 0.3048 | 0.00161 |
| ESA_00701 | A7MGZ3 | Uncharacterized protein | 0.2999 | 0.00179 |
| ESA_02217 | A7MFU1 | Uncharacterized protein | 0.2997 | 0.00113 |
| rpmJ1 | A7MPF5 | 50S ribosomal protein L36 1 | 0.2981 | 0.02615 |
| diaA | A7MIP9 | DnaA initiator-associating protein DiaA | 0.2979 | 0.00786 |
| ESA_00796 | A7MKY2 | Uncharacterized protein | 0.2973 | 0.01591 |
| ESA_02956 | A7MLS1 | Uncharacterized protein | 0.2967 | 0.00203 |
| ESA_03488 | A7MIT4 | Uncharacterized protein | 0.2946 | 0.02454 |
| cysS | A7MK00 | Cysteine--tRNA ligase | 0.2925 | 0.00022 |
| ESA_03633 | A7MJB2 | Uncharacterized protein | 0.2920 | 0.00390 |
| purL | A7MGZ6 | Phosphoribosylformylglycinamidine synthase | 0.2915 | 0.00076 |
| ESA_01653 | A7MGH4 | UPF0283 membrane protein ESA_01653 | 0.2893 | 0.00345 |
| ESA_03502 | A7MIR7 | Uncharacterized protein | 0.2856 | 0.00769 |
| ESA_00480 | A7MR29 | Uncharacterized protein | 0.2875 | 0.00202 |
| ESA_03450 | A7MIU2 | Uncharacterized protein | 0.2874 | 0.00006 |
| ESA_01090 | A7MLJ2 | Uncharacterized protein | 0.2872 | 0.01271 |
| ESA_00318 | A7MLZ1 | Citrate-sodium symporter | 0.2858 | 0.00364 |
| ESA_02423 | A7MEU8 | Uncharacterized protein | 0.2842 | 0.00000 |
| ESA_02357 | A7MFZ1 | Bifunctional protein PutA | 0.2835 | 0.00011 |
| ESA_00208 | A7MM69 | Uncharacterized protein | 0.2835 | 0.01111 |
| ESA_04188 | A7MKQ8 | Uncharacterized protein | 0.2825 | 0.00592 |
| pqqC | A7MN58 | Pyrroloquinoline-quinone synthase | 0.2814 | 0.00446 |
| ESA_00380 | A7MJS5 | Uncharacterized protein | 0.2781 | 0.00501 |
| ESA_00785 | A7ML02 | Uncharacterized protein | 0.2751 | 0.00768 |
| ESA_01187 | A7MHD6 | Uncharacterized protein | 0.2747 | 0.00010 |
| ESA_03296 | A7MIA5 | Uncharacterized protein | 0.2741 | 0.00616 |
| ESA_04064 | A7MQD7 | Uncharacterized protein | 0.2738 | 0.00017 |
| plsY | A7MJT5 | Glycerol-3-phosphate acyltransferase | 0.2725 | 0.03563 |
| ESA_00963 | A7MHS3 | Uncharacterized protein | 0.2714 | 0.00391 |
| ESA_03609 | A7MJC9 | Uncharacterized protein | 0.2664 | 0.00154 |
| lgt | A7MR30 | Prolipoprotein diacylglyceryl transferase | 0.2653 | 0.00679 |
| ESA_01854 | A7MK93 | Uncharacterized protein | 0.2643 | 0.00885 |
| ESA_03948 | A7MPZ5 | Uncharacterized protein | 0.2607 | 0.02953 |
| ESA_00284 | A7MNC9 | Uncharacterized protein | 0.2592 | 0.00046 |
| gppA | A7MQI3 | Guanosine-5'-triphosphate,3'-diphosphate pyrophosphatase | 0.2590 | 0.00543 |
| ESA_02131 | A7MNX1 | Uncharacterized protein | 0.2580 | 0.00064 |
| ESA_02461 | A7MER2 | Uncharacterized protein | 0.2549 | 0.00820 |
| clpS | A7MEQ8 | ATP-dependent Clp protease adapter protein ClpS | 0.2546 | 0.03724 |
| ESA_02248 | A7MFR3 | Uncharacterized protein | 0.2541 | 0.0052 |
| ESA_03928 | A7MQ23 | Uncharacterized protein | 0.2531 | 0.00097 |
| ESA_00613 | A7MEH4 | Uncharacterized protein | 0.2525 | 0.00343 |
| ESA_03407 | A7MG87 | Uncharacterized protein | 0.2506 | 0.00068 |
| ESA_01443 | A7MKF1 | Uncharacterized protein | 0.2484 | 0.00023 |
| ESA_03112 | A7MEM9 | Uncharacterized protein | 0.2458 | 0.00326 |
| glnE | A7MP92 | Bifunctional glutamine synthetase adenylyltransferase/adenylyl-removing enzyme | 0.2395 | 0.01730 |
| ESA_01908 | A7MMS1 | Uncharacterized protein | 0.2362 | 0.02864 |
| zapE | A7MJC2 | Cell division protein ZapE | 0.2327 | 0.00727 |
| ESA_03947 | A7MQ11 | Uncharacterized protein | 0.2202 | 0.00112 |
| ESA_00429 | A7MR87 | Uncharacterized protein | 0.2103 | 0.00004 |
| ESA_01236 | A7MJL0 | Uncharacterized protein | 0.2102 | 0.00090 |
| ESA_01288 | A7MJI4 | Flagellin | 0.2081 | 0.00756 |
| ESA_01236 | A7MJL0 | Uncharacterized protein |  |  |
| ESA_00459 | A7MR50 | Uncharacterized protein | 0.2051 | 0.00009 |
| ESA_01295 | A7MP25 | Uncharacterized protein | 0.2048 | 0.03505 |
| ESA_02532 | A7MEZ0 | Uncharacterized protein | 0.2033 | 0.00022 |
| ESA_03854 | A7ML50 | Alpha-galactosidase | 0.2032 | 0.00060 |
| ESA_00975 | A7MHS1 | FAD:protein FMN transferase | 0.2028 | 0.01420 |
| kup | A7MMV3 | Low affinity potassium transport system protein kup | 0.2002 | 0.00229 |
| ESA_02734 | A7MNP1 | Uncharacterized protein | 0.1967 | 0.00283 |
| ESA_01892 | A7MK45 | Uncharacterized protein | 0.1962 | 0.00026 |
| ESA_02227 | A7MFT6 | Uncharacterized protein | 0.1935 | 0.00036 |
| uxaC | A7MIR9 | Uronate isomerase | 0.1912 | 0.00001 |
| yidC | A7MN02 | Membrane protein insertase YidC | 0.1891 | 0.02726 |
| ESA_01768 | A7MLB5 | Uncharacterized protein | 0.1879 | 0.00195 |
| ESA_02242 | A7MFS2 | Uncharacterized protein | 0.1849 | 0.00008 |
| cmoM | A7MEU6 | tRNA 5-carboxymethoxyuridine methyltransferase | 0.1851 | 0.00621 |
| ESA_02178 | A7MNT3 | Uncharacterized protein | 0.1823 | 0.02742 |
| ESA_01519 | A7MMJ8 | Uncharacterized protein | 0.1808 | 0.00366 |
| ESA_03362 | A7MIG2 | Uncharacterized protein | 0.1801 | 0.00350 |
| ESA_01617 | A7MGL2 | Uncharacterized protein | 0.1743 | 0.01427 |
| ESA_01468 | A7MKD1 | Uncharacterized protein | 0.1738 | 0.00001 |
| ESA_04208 | A7MKP3 | Uncharacterized protein | 0.1737 | 0.00821 |
| ESA_03131 | A7MI39 | Uncharacterized protein | 0.1730 | 0.00060 |
| ESA_pESA3p05527 | A7MRM1 | Uncharacterized protein | 0.1682 | 0.00954 |
| ESA_02629 | A7MQW7 | Uncharacterized protein | 0.1650 | 0.00000 |
| ESA_04076 | A7MQB2 | Uncharacterized protein | 0.1590 | 0.01884 |
| ESA_01083 | A7MLK0 | S-formylglutathione hydrolase | 0.1550 | 0.00002 |
| folE | A7MLK2 | GTP cyclohydrolase 1 | 0.1535 | 0.00670 |
| ESA_03478 | A7MQF7 | Uncharacterized protein | 0.1531 | 0.00003 |
| ESA_01583 | A7MME9 | Uncharacterized protein | 0.1490 | 0.00130 |
| ESA_00861 | A7MKU1 | Uncharacterized protein | 0.1435 | 0.00308 |
| ESA_01082 | A7MLJ9 | Uncharacterized protein | 0.1393 | 0.00692 |
| ESA_00878 | A7MH77 | Uncharacterized protein | 0.1392 | 0.00540 |
| ESA_02895 | A7MLX7 | Uncharacterized protein | 0.1389 | 0.01140 |
| ESA_00340 | A7MJV4 | Uncharacterized protein | 0.1373 | 0.01113 |
| ESA_03921 | A7MQ33 | Uncharacterized protein | 0.1267 | 0.01758 |
| ESA_01838 | A7MPQ9 | Anti-sigma factor antagonist | 0.1188 | 0.00056 |
| ESA_02959 | A7MLS5 | Carbonic anhydrase | 0.1152 | 0.00081 |
| ESA_00862 | A7MKU2 | Uncharacterized protein | 0.1126 | 0.00025 |
| dusC | A7MHN0 | tRNA-dihydrouridine synthase | 0.1105 | 0.03095 |
| ESA_02022 | A7MFB8 | Uncharacterized protein | 0.1085 | 0.00366 |
| ESA_04186 | A7MNI0 | Uncharacterized protein | 0.1050 | 0.02024 |
| ESA_01876 | A7MK64 | Uncharacterized protein | 0.0965 | 0.02326 |
| nanA | A7MJD2 | N-acetylneuraminate lyase | 0.0810 | 0.00079 |
| ESA_00928 | A7MH33 | 5'-deoxynucleotidase ESA_00928 | 0.0729 | 0.00761 |
| ESA_00610 | A7MEH1 | Uncharacterized protein | 0.0660 | 0.00665 |
| ESA_02422 | A7MEU7 | Uncharacterized protein | 0.2842 | 0.00361 |
| ESA_00614 | A7MHY9 | Uncharacterized protein | 0.0527 | 0.00366 |
| ESA_04343 | A7MGB7 | Uncharacterized protein | 0.0518 | 0.00420 |
| ESA_02967 | A7MLR8 | Uncharacterized protein | 0.0287 | 0.00621 |
| ESA_01889 | A7MK59 | Uncharacterized protein | 0.0158 | 0.00070 |
| mtgA | A7MJD8 | Biosynthetic peptidoglycan transglycosylase | 0.0014 | 0.00000 |
| ESA_01125 | A7MHK7 | Uncharacterized protein | 0.0008 | 0.00178 |
| ESA_02806 | A7MJW2 | Uncharacterized protein | 0.0006 | 0.01175 |
| ESA_02488 | A7MF23 | Putative transport protein ESA_02488 | 0.0004 | 0.02729 |
| ESA_01099 | A7MHN1 | Uncharacterized protein | 0.0003 | 0.01850 |
| ESA_02855 | A7MFK0 | Uncharacterized protein | 0.0002 | 0.00540 |
| ESA_00387 | A7MP89 | UPF0235 protein ESA_00387 | 0.0002 | 0.00010 |
| ESA_00612 | A7MEH3 | Uncharacterized protein | 0.0002 | 0.02231 |
| ESA_01830 | A7MPR8 | Uncharacterized protein | 0.0001 | 0.00168 |
| ESA_03437 | A7MG53 | Uncharacterized protein | 0.0001 | 0.00734 |
| ESA_03294 | A7MIA1 | Uncharacterized protein | 0.0001 | 0.02607 |
| ESA_02902 | A7MEM0 | Uncharacterized protein | 0.0001 | 0.03772 |
| ESA_00706 | A7MGZ7 | Uncharacterized protein | 0.0001 | 0.00133 |
| ESA_01837 | A7MPQ8 | Uncharacterized protein | 0.0001 | 0.00055 |
| ESA_02908 | A7MLW7 | Uncharacterized protein | 0.0001 | 0.00032 |
| nagK | A7MFT5 | N-acetyl-D-glucosamine kinase | 0.0001 | 0.02332 |
| ESA_02142 | A7MNW3 | Uncharacterized protein | 0.0001 | 0.00287 |
| ESA_03306 | A7MIL7 | Uncharacterized protein | 0.0001 | 0.00034 |

“∞” indicates that the proteins are only detected in the strongly adhesive/invasive strain SAKA80220.

Abundance ratios ≥3 and P value ≤0.05 indicates that the proteins were expressed higher in *C. sakazakii* SAKA80220 (strongly adhesive/invasive strain) compared with *C. sakazakii* SAKA80221 (weakly adhesive/invasive strain).

Abundance ratios ≤0.33 and P value ≤0.05 indicates that the proteins were expressed lower in *C. sakazakii* SAKA80220 (strongly adhesive/invasive strain) compared with *C. sakazakii* SAKA80221 (weakly adhesive/invasive strain).
